# Children of the Church

**Published:** 1871-09

**Authors:** 


### CHILDREN OF THE CHURCH,

For whom the Church should be bound to provide, are clergymen who have become helpless in the service of the Church; they, with their wives and helpless children, have a claim to the support of the Church which should be honored as a matter of Christian principle. Those who need argument to prove this, could scarcely be convinced by any array of facts which could be presented; but to the intelligent and generous hearted of all denominations of Christian people, the example of the "world's people" is well worthy of imitation. One of the most honored of Massachusetts' sons died suddenly not long ago, leaving his family destitute; a few political friends secured an amount of money, the interest of which will be a permanent comfort to the widow and little ones. A cabinet officer died in poverty last year, and in a very short time a hundred thousand dollars were raised for his widow; another eminent name in the nation's history has been honored with a people's remembrance by large and liberal contributions to the dear ones left behind him. The men who have given their time, their talents, their energies, and their life to the Church are surely as much entitled to the Church's remembrance as those who have given themselves to the service of the State, with not half the entire consecration to their work which characterizes clergymen. Not long ago a clergyman died who for forty years had been a preacher of the gospel, and whose receipts for his work averaged a hundred dollars a year. Within a few months an old minister's friends appealed to the Church for a little money to pay off a mortgage on the old man's humble home; the four hundred dollars needed for that purpose removed an insupportable weight from his mind, and made him the happiest of men. Last year the papers reported that a helpless old clergyman was sold in Pennsylvania to the lowest bidder; that is, the man who would take care of him for the smallest sum should have the bargain. It is to the ministers of the gospel that the women of America owe their elevation; it is to the ministers of the gospel this nation owes it that it is not a French Commune, and thinking of this, let every church raise a "Disabled Ministers' Fund," the interest of which will amply provide for helpless ministers and their families.

